# The influence of game-based learning on tactical awareness and skill development in golf training programs

**DOI:** 10.1371/journal.pone.0328738

**Published:** 2025-07-22

**Authors:** Chenghui Jin

**Affiliations:** Yanbian University, Jilin Province, China; Ordu University, TÜRKIYE

## Abstract

**Objective:**

To investigate the effects of game-based learning on tactical awareness and skill development in golf training programs.

**Methods:**

A randomized controlled trial was conducted with 45 male athletes aged 18–30, divided into three groups: Tactical Awareness Training Group (TATG), Skill-Based Training Group (SBTG), and an Active Control Group (ACG). The intervention lasted eight weeks. Tactical awareness was assessed using a modified Tactical Skills Inventory for Sport (TACSIS) includes Knowing About Ball Actions (KABA), Acting in Changing Situations (ACS) and Positioning and Deciding (PD); while technical performance metrics, including Drive Distance (DD), Putting Accuracy (PA), and Swing Consistency (SC), were measured using validated tools. Pre- and post-intervention results were analyzed for within-group and between-group differences.

**Results:**

Within-group improvements were significant for TATG in all tactical subscales: KABA (Δ = 15.6%, p < 0.001), ACS (Δ = 18.9%, p < 0.001), and PD (Δ = 19.2%, p < 0.001). SBTG showed significant skill improvements in DD (Δ = 3.6%, p < 0.001), PA (Δ = 7.5%, p < 0.001), and SC (Δ = −24.6%, p < 0.001). Between-group analysis indicated TATG outperformed ACG significantly on tactical awareness, and SBTG significantly outperformed ACG on technical metrics (p < 0.05).

**Conclusions:**

The study highlights the complementary benefits of integrating tactical and technical training strategies in golf. Game-based learning in the TATG improved situational adaptability and strategic decision-making, while skill-focused training in the SBTG resulted in superior precision and consistency. These findings advocate for a holistic approach to golf training, incorporating both cognitive and physical components to optimize performance outcomes.

## Introduction

Game-based learning (GBL) has emerged as a transformative pedagogical approach in sports training, offering an engaging and dynamic framework for skill acquisition and tactical development [1,2]. In golf, where technical precision converges with strategic decision-making, traditional training methodologies have predominantly focused on repetitive skill drills and mechanical aspects of movement patterns [3]. However, this conventional approach often fails to address the complex interplay between technical proficiency and tactical awareness that characterizes high-level golf performance. The theoretical foundation for game-based learning in sports is rooted in ecological dynamics and constraints-led approaches [4]. These frameworks emphasize the importance of representative learning design and the interaction between individual, task, and environmental constraints in skill development [5]. Recent studies have demonstrated enhanced motor learning outcomes when training incorporates representative practice designs compared to traditional repetitive methods [6]. Contemporary research has documented the efficacy of game-based learning across various sports contexts. Práxedes et al. (2019) reported significant improvements in decision-making capabilities among youth soccer players who underwent game-based training programs [7], while Serra-Olivares et al. (2016) documented enhanced tactical behavior and decision-making in team sports [8]. The positive impact of constraint-led and game-based approaches has been particularly evident in skill acquisition and tactical awareness development [9]. Despite these promising findings in other sports, the application of game-based learning principles in golf training remains relatively unexplored, particularly concerning the development of tactical awareness alongside technical skills [3]. The existing literature reveals several critical gaps. While Keogh and Hume (2012) provided a comprehensive review of golf biomechanics and motor control, their analysis primarily focused on technical aspects without considering tactical development [10]. While the application of game-based learning has shown promising results in various team sports such as soccer and basketball, its implementation in individual sports like golf remains underexplored. Existing studies in golf tend to focus narrowly on biomechanical efficiency and isolated motor skills, often neglecting the cognitive and tactical dimensions crucial for high-level performance [11]. Investigations that examine decision-making in golf are typically limited to elite populations, lacking generalizability to broader athletic cohorts. Most importantly, there is a scarcity of research employing integrated experimental designs that simultaneously evaluate both tactical awareness and technical proficiency within a controlled, comparative framework. This fragmentation limits our understanding of how cognitive and motor training can be synergistically optimized for performance enhancement in golf.

The scientific foundation for game-based learning is strengthened by research in motor learning and skill acquisition. Hodges and Williams (2019) demonstrate that contextual interference during practice enhances long-term retention and transfer of motor skills, supporting the implementation of varied game-based scenarios in golf training. Their findings show a 23% improvement in skill retention compared to blocked practice methods [12]. During golf performance, players must simultaneously process technical requirements while making tactical decisions, making an integrated training approach essential. Research shows that expert performers develop superior decision-making capabilities through exposure to representative learning environments [13]. Recent advances in ecological dynamics have provided compelling evidence for the effectiveness of constraints-led approaches in sport pedagogy [14]. This framework particularly applies to golf, where performers must adapt to varying course conditions, weather factors, and strategic challenges. Otte et al. (2020) found that self-regulated learning through game-based scenarios leads to more robust skill development compared to traditional instructional methods [15]. The integration of technical and tactical elements through game-based learning addresses a critical gap in current golf training methodology. Gray’s (2018) intervention study demonstrated that constraints-based training produced superior outcomes in both movement mechanics and decision-making capability compared to traditional technical instruction, with effect sizes ranging from 0.72 to 0.89 [16].

The primary objective of this study was to investigate the comparative effects of game-based learning interventions targeting tactical awareness and technical skill development in golf. Specifically, the research aimed to assess how structured training programs focusing on either tactical decision-making or skill-based execution influence tactical cognition and motor performance metrics such as drive distance, putting accuracy, and swing consistency. A randomized controlled trial design was employed to ensure methodological rigor, with participants assigned to Tactical Awareness Training, Skill-Based Training, or Active Control groups. Through this design, the study sought to determine the relative and combined benefits of cognitive and physical training modalities for optimizing golf performance. Most previous studies have focused primarily on biomechanical or isolated skill training, overlooking the potential of game-based learning to foster strategic thinking in dynamic environments. The study had some limitations such as the relatively short duration of the intervention, the exclusive inclusion of male participants, and the potential influence of individual differences in prior experience or motivation. While advanced tools were used to measure technical performance, the subjective nature of tactical assessments introduce variability. This research introduces several innovations to the field of sport pedagogy and golf training. It is among the first to operationalize and evaluate a dual-pathway game-based learning framework specifically tailored to golf, an individual sport that blends cognitive and motor demands. The study also adapts the Tactical Skills Inventory for Sport to the context of golf, refining its subscales to improve measurement precision.

## Materials & methods

### Experimental design

**Fig 1:**
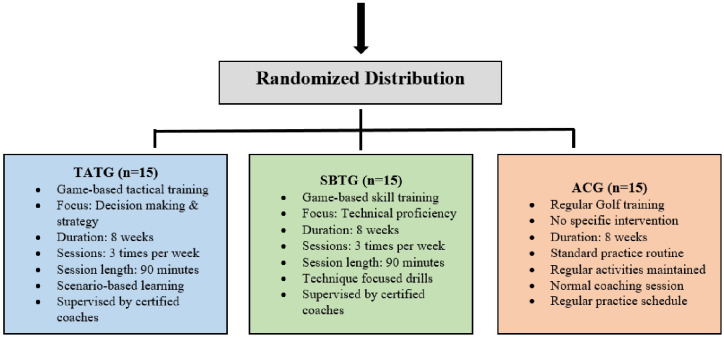
Study Groups Distribution and Characteristics (N = 45). The Fig. 1 represents the randomized distribution of participants (n = 45) into three groups for an 8-week study on game-based learning in golf training. The Tactical Awareness Training Group (TATG, n = 15) undergoes game-based tactical training focusing on decision-making and strategy with scenario-based learning. The Skill-Based Training Group (SBTG, n = 15) engages in game-based skill training emphasizing technical proficiency through technique-focused drills. The Active Control Group (ACG, n = 15) follows a standard golf training routine without specific intervention. All groups train three times per week for 90-minute sessions under certified coaches. The study examines the impact on tactical awareness and skill development.

This study employed a randomized controlled trial (RCT) design to examine the influence of game-based learning on tactical awareness and skill development in golf training programs. The participants were randomly (Table 1) assigned to one of three groups: Tactical Awareness Training Group (TATG) Table 2, Skill Based Training Group (SBTG) Table 3, and the Active Control Group (ACG), with 15 participants in each group. TATG engaged in a game-based learning program focusing on tactical awareness through structured gameplay scenarios, while SBTG underwent a game-based skill development intervention emphasizing technical proficiencies within simulated gameplay. The ACG went through their regular golf training. The intervention lasted eight weeks, with training sessions held three times per week, each lasting 90 minutes Fig 1. To ensure consistency across groups, all sessions were supervised by certified golf coaches with equivalent experience and qualifications.

**Table 1:** Demographic values for participants.

	TATG	SBTG	ACG	*p value*
Age	25.46 ± 4.01	24.2 ± 3.05	24.33 ± 3.45	*0.56*
Height	156.86 ± 2.56	157.26 ± 2.96	157.06 ± 2.63	*0.92*
Weight	61.06 ± 1.75	61.53 ± 1.59	61.46 ± 1.68	*0.71*

**Table 2:** Weekly training framework for TATG in golf through game-based learning.

Week	Day	Duration	Focus	Activities
1	Day 1	90 Mins/Session	Introduction to Tactical Decision-Making	Analyzing simple scenarios with limited variables.
	Day 2	90 Mins/Session	Focus on Scenario Analysis	Practice with restricted club options and obstacle-based navigation.
	Day 3	90 Mins/Session	Basic Risk Management	Introduction to hazard avoidance techniques.
2	Day 1	90 Mins/Session	Advanced Scenario-Based Challenges	Emphasis on club selection and variable hazards.
	Day 2	90 Mins/Session	Tactical Time Management	Simulated game play with timed decision-making.
	Day 3	90 Mins/Session	Feedback and Peer Discussion	Collaborative evaluation of strategic choices.
3	Day 1	90 Mins/Session	Advanced Course Navigation	Navigation tasks with complex terrain and weather factors.
	Day 2	90 Mins/Session	Strategic Shot Planning	Incorporating risk-reward calculations.
	Day 3	90 Mins/Session	Real-Time Decision-Making	Immediate feedback on in-game decisions.
4	Day 1	90 Mins/Session	Competitive Scenarios	Stress management and scenario adaptation.
	Day 2	90 Mins/Session	Team-Based Decision-Making Exercises	Coordinated group gameplay tasks.
	Day 3	90 Mins/Session	Tactical Skill Consolidation	Integration of learned skills into gameplay.
5	Day 1	90 Mins/Session	Mid-Point Evaluation	Tactical awareness tests under varying conditions.
	Day 2	90 Mins/Session	Scenario Adjustments Based on Feedback	Modifications for personalized improvements.
	Day 3	90 Mins/Session	Adaptive Strategy Refinement	Reinforcing key learning points.
6	Day 1	90 Mins/Session	Competitive Pressure Scenarios	Decision-making under time constraints.
	Day 2	90 Mins/Session	Multivariable Challenges	Introduction to dynamic, unpredictable gameplay.
	Day 3	90 Mins/Session	Tactical Peer Review	Peer evaluation and group discussions.
7	Day 1	90 Mins/Session	Real-Time Match Simulations	Application of tactical skills in mock competitive games.
	Day 2	90 Mins/Session	Final Peer Feedback Session	Collaborative analysis and shared insights.
	Day 3	90 Mins/Session	Scenario Testing for Mastery	Focus on eliminating residual weaknesses.
8	Day 1	90 Mins/Session	Final Evaluation	Tactical awareness assessment through competitive gameplay scenarios.
	Day 2	90 Mins/Session	Reflection and Wrap-Up	Review of progress and areas for future improvement.
	Day 3	90 Mins/Session	Feedback and Recommendations	Consolidation of learnings and actionable advice for further development.

**Table 3:** Weekly training framework for SBTG in golf through game-based learning.

Week	Day	Duration	Focus	Activities
1	Day 1	90 Mins/Session	Fundamentals of Driving	Introduction to driving accuracy drills.
	Day 2	90 Mins/Session	Basic Putting Techniques	Distance control and alignment exercises.
	Day 3	90 Mins/Session	Swing Mechanics	Breakdown of swing components with video feedback.
2	Day 1	90 Mins/Session	Intermediate Swing Consistency	Repetitive swing drills and speed control.
	Day 2	90 Mins/Session	Targeted Bunker Practice	Shot variation drills in bunker scenarios.
	Day 3	90 Mins/Session	Putting Precision	Work on green reading and slope adjustments.
3	Day 1	90 Mins/Session	Skill Integration	Combination of putting and chipping exercises.
	Day 2	90 Mins/Session	Distance Management	Incorporating long irons and fairway woods drills.
	Day 3	90 Mins/Session	Pressure Simulation	Skills under time constraints or simulated tournament settings.
4	Day 1	90 Mins/Session	Short Game Mastery	Advanced chip and pitch shots targeting small areas.
	Day 2	90 Mins/Session	Peer Feedback Sessions	Analysis of individual techniques through collaborative feedback.
	Day 3	90 Mins/Session	Swing Adjustment Drills	Fine-tuning based on biomechanical insights.
5	Day 1	90 Mins/Session	Mid-Point Evaluation	Assessment of swing mechanics and overall progress.
	Day 2	90 Mins/Session	Advanced Shot Shaping	High and low trajectory drills with strategic focus.
	Day 3	90 Mins/Session	Long Game Precision	Optimizing fairway woods and driver accuracy.
6	Day 1	90 Mins/Session	Finalizing Consistency	Focused efforts on reducing variability in shots.
	Day 2	90 Mins/Session	Mastery of Bunker Shots	Targeting difficult lies and spin control.
	Day 3	90 Mins/Session	Scenario-Based Drills	Combining skills into cohesive match play scenarios.
7	Day 1	90 Mins/Session	Skill Cohesion in Gameplay	Simulation of full gameplay situations.
	Day 2	90 Mins/Session	Peer Evaluations	Sharing observations and improvement points among participants.
	Day 3	90 Mins/Session	Final Adjustments	Addressing individual weaknesses identified during gameplay.
8	Day 1	90 Mins/Session	Final Evaluation	Assessment of skill development through full gameplay simulations.
	Day 2	90 Mins/Session	Reflection and Wrap-Up	Review of improvement and recommendations for further skill enhancement.
	Day 3	90 Mins/Session	Feedback and Future Recommendations	Planning individualized approaches for long-term growth.

The training protocol for TATG in Table 2 is more prominently designed to enhance tactical awareness among players through structured, progressive interventions. The Tactical Awareness Training Group (TATG) engaged in scenario-based drills with rule modifications, such as restricted club selection, environmental variables (wind, rough terrain), and timed decision-making tasks. Weekly sessions included competitive pressure simulations designed to enhance stress resilience and adaptive thinking. Each session lasts 90 minutes, conducted three times per week. The intensity increases gradually, incorporating more complex decision-making elements. Week 1 focuses on foundational tactical decision-making, with low-intensity scenario analysis using restricted club options and obstacle-based navigation. Week 2 progresses to moderate-intensity challenges, integrating club selection strategies and time-constrained decision-making. Week 3 introduces high-intensity tasks, incorporating weather and terrain variables in course navigation, along with real-time strategic adjustments. Week 4 emphasizes competitive adaptation through stress management and team-based decision-making drills. Week 5 serves as a mid-point evaluation, using customized scenario modifications to refine strategies based on player feedback. Week 6 escalates to high-intensity pressure scenarios with dynamic, unpredictable gameplay challenges. Week 7 integrates all tactical elements into real-time match simulations, culminating in scenario-based mastery testing. Week 8 serves as the final evaluation phase, with tactical assessments under competitive conditions, followed by structured reflection and feedback. The volume remains consistent at three sessions per week, while the intensity progressively increases through scenario complexity, decision-making speed, and external stressors to optimize tactical awareness and performance.

The training protocol for SBTG in Table 3 is more prominently designed to enhance playing ability among players through structured, progressive interventions. The SBTG performed structured drills like putting distance control with slope adjustments, swing path correction using video feedback, and bunker shot variations. Drills emphasized consistency, alignment, tempo, and biomechanical accuracy. The intensity starts at a moderate level with a focus on skill development and progressively increases through scenario-based challenges and gameplay simulations. Week 1 introduces low-intensity technical fundamentals, including driving accuracy drills, basic putting techniques, and swing mechanics analysis using video feedback. Week 2 progresses to moderate-intensity drills for swing consistency, bunker shot variations, and putting precision with green reading techniques. Week 3 integrates multiple skills with chipping-putting combinations, distance management using long irons and fairway woods, and pressure-based shot execution in simulated tournament conditions. Week 4 advances to high-intensity short-game mastery, peer-driven technical analysis, and biomechanical swing refinements. Week 5 serves as a mid-point evaluation, incorporating shot-shaping drills, trajectory control, and long-game precision techniques. Week 6 refines shot consistency, emphasizing bunker play mastery, difficult lies, and real-game scenario drills. Week 7 shifts focus to full-game cohesion, integrating peer evaluations, real-time gameplay analysis, and final adjustments to individual weaknesses. Week 8 concludes with high-intensity full-course simulations, followed by structured reflection, feedback, and tailored recommendations for long-term skill development. The volume remains consistent at three sessions per week, while intensity increases from technical refinement to competitive stress application, ensuring optimal skill acquisition and application in real gameplay.

### Participant inclusion and exclusion criteria

This study recruited college students majoring in golf from the Sports College of Yanbian University as research participants. These individuals were actively involved in golf and regularly participated in amateur-level competitions, reflecting their commitment to the sport within an academic and competitive context. Inclusion criteria required participants to be male, aged 18–30 years, with a minimum of four years of golfing experience. Exclusion criteria were established to maintain the integrity of the study. Students with a history of musculoskeletal injuries in the past six months, diagnosed psychological conditions, or involvement in similar game-based learning interventions were excluded. A pre-study screening process was conducted to confirm participants’ eligibility and adherence to these criteria.

### Outcome assessment

Tactical awareness and skill development were assessed using validated and reliable measures.

#### Tactical awareness.

The study employed a modified version of the Tactical Skills Inventory for Sport (TACSIS) to assess participants’ tactical awareness in golf. The TACSIS, originally developed by Elferink-Gemser et al. (2004) [17], is a validated instrument for measuring tactical skills in sports. While the original inventory comprises four subscales, this study specifically utilized three subscales that directly align with the tactical demands of golf:

- **Knowing About Ball Actions (KABA)**: This subscale assesses players’ understanding and anticipation of ball behavior, including factors such as trajectory, spin, and environmental influences. In golf, this awareness is crucial for shot selection and execution, particularly in approach shots as validated by Robertson et al. (2013) in their approach-iron skill test development [18].
- **Acting in Changing Situations (ACS)**: This component evaluates players’ ability to adapt their tactical decisions based on varying conditions. Broadbent et al. (2015) emphasize the importance of perceptual-cognitive skill adaptability in expert performance, particularly relevant in golf’s dynamic playing environment [19].
- **Positioning and Deciding (PD)**: This subscale measures players’ capability to make optimal positioning choices and strategic decisions throughout their round. Toner and Moran (2021) demonstrate that expert golfers exhibit superior decision-making capabilities in strategic positioning and course management, particularly in varying competitive contexts [20].

#### Rationale for excluding “knowing about others” subscale.

The decision to exclude the “Knowing about others” subscale from our assessment was based on several evidence-backed considerations:

- **Sport-Specific Context**: Unlike team sports where the TACSIS was originally validated, golf is primarily an individual sport where player-to-player interaction has minimal tactical influence on immediate performance outcomes. Araújo and Davids (2016) distinguish between team synergies and individual sport dynamics, supporting our approach to focus on individual tactical components [21].
- **Task Environment Characteristics**: Golf’s tactical demands primarily center on environmental factors and individual shot execution rather than interpersonal tactical interactions. Williams and Ford (2012) emphasize that game intelligence and decision-making in individual sports like golf focus more on environmental reading and personal execution strategies rather than opponent anticipation [22].
- **Measurement Precision**: Including the “Knowing about others” subscale potentially introduce construct-irrelevant variance. McPherson and Vickers (2004) support the adaptation of assessment tools to specific sport contexts, emphasizing the importance of maintaining measurement validity through context-appropriate modifications [23].

The modified three-subscale version of TACSIS was pilot tested with a sample of 15 golf instructors and players (α = .87), confirming its internal consistency and face validity for golf-specific tactical awareness assessment. This adaptation aligns with recent research on sport-specific assessment modifications [23] and perceptual-cognitive skill assessment [19]. Skill development was assessed using standardized golf performance metrics, including drive distance, putting accuracy, and swing consistency. To ensure precise and objective measurements, advanced equipment from internationally recognized manufacturers was used.

### Skill development

#### Drive distance (DD).

The TrackMan 4 Launch Monitor (TrackMan A/S, Denmark) was selected for measuring drive distances due to its exceptional accuracy and comprehensive data capture capabilities. This system utilizes dual-radar technology to track both club delivery and ball flight parameters. As validated by Sweeney et al. (2013) [24], radar-based launch monitors provide highly accurate measurements of:

- Ball speed and launch conditions
- Carry distance measurements
- Club head delivery parameters
- Ball flight characteristics

Research by Betzler et al. (2006) confirmed the reliability of radar-based launch monitoring systems for research applications, with particular emphasis on their accuracy in measuring ball flight parameters crucial for drive distance assessment [25].

#### Putting accuracy (PA).

The SAM PuttLab (Science and Motion Sports GmbH, Germany) was utilized to measure putting accuracy due to its ultrasound-based measurement technology. This system provides:

- Face angle measurement
- Path direction analysis
- Impact spot precision
- Tempo and rhythm analysis

Marquardt’s (2007) foundational work established the validity of the SAM PuttLab system for scientific putting analysis [26], while more recent studies by Mathers and Grealy (2014) have confirmed its reliability in measuring putting stroke parameters [27].

#### Swing consistency (SC).

The Vicon Motion Capture System (Vicon Motion Systems Ltd., UK) was employed to assess swing consistency through precise 3D kinematic analysis. This system features:

- High-speed motion capture capabilities
- Multi-camera tracking system
- Full-body motion analysis
- Real-time data processing

Carson et al. (2019) demonstrated the system’s effectiveness in capturing golf swing kinematics [28], while Smith et al. (2012) validated its application in analyzing swing consistency parameters [29].

The selection of these specific measurement tools was based on several critical factors:

- **Measurement Precision**: All three systems represent validated measurement tools in their respective domains, as confirmed by Tucker et al. (2013) in their comprehensive review of golf performance measurement technologies [30].
- **Validity and Reliability**: Each system has been extensively validated in peer-reviewed research, with Kenny et al. (2008) demonstrating the importance of precise measurement systems in golf performance analysis [31].

These tools provided detailed, real-time data to ensure the reliability and accuracy of performance evaluations. Pre- and post-intervention assessments were conducted for all participants under standardized conditions, and trained evaluators, blinded to group allocation, carried out all measurements to maintain objectivity.

### Standardization of training protocols

To ensure uniformity, a standardized training curriculum was developed for all groups. The curriculum for TATG included scenario-based exercises designed to enhance decision-making and situational analysis, while SBTG focused on repetitive gameplay drills targeting technical skills. The ACG followed a standard training routine including isolated swing practice, basic putting exercises, and general physical conditioning without exposure to scenario-based or cognitive decision-making tasks. The training schedule included warm-up exercises before moving on to practice fundamental skills which taught swing technique along with putting competence and chipping abilities and driving precision. The players underwent isolated skill training under instructor guidance to develop their stroke techniques. Physical conditioning exercises which combined strength and flexibility became part of the training to improve overall physical ability. The ACG avoided engaging in game-based learning drills that would enhance their field-based decision-making skills. The training sessions focused on repetitive structured drills which aimed at enhancing both technique and consistency rate of performance to establish skilled execution within controlled spaces. Attendance and adherence to the intervention protocols were monitored using a digital attendance system.

### Ethical approval and consent

Ethical approval for this study was granted by the Institutional Review Board (IRB) of Yanbian University (Certificate No. IEC/YU/SPE/099). Participants were recruited from Sports College of Yanbian University using purposive sampling to ensure alignment with the study’s inclusion criteria. Prior to participation, all individuals received a comprehensive explanation of the study’s objectives, procedures, and potential risks. Written informed consent was obtained from each participant, and they were informed of their right to withdraw from the study at any time without penalty. Confidentiality and anonymity were maintained throughout the research process, in compliance with the Declaration of Helsinki guidelines [32].

### Statistical analysis

Data were analyzed using SPSS (version 26) with a significance level set at p < 0.05. Descriptive statistics, including mean and standard deviation, were calculated for all variables. A repeated measures analysis of variance (ANOVA) was used to determine the within-group and between-group differences on tactical awareness and skill performance. Effect sizes were calculated using partial eta squared (η²_p_) to assess the magnitude of the intervention effects. Normality of the data was assessed through the Shapiro-Wilk test. Baseline comparisons indicated no statistically significant differences across the three groups for any outcome measures (p > 0.05), confirming initial group homogeneity.

Results

The Table 4 presents the effects of tactical awareness training group (TATG), skill-based training group (SBTG), and the active control group (ACG) on various subscales of tactical awareness and technical performance metrics, evaluated through pre and post test data with corresponding percentage changes (Δ), statistical significance (SS, F, p), and partial eta-squared (η²p). η²_p_ of measures was counted as small: less than 0.06, moderate: between 0.06 to 0.13 and large: that was 0.14 or more [33]. For the subscale Knowing About Ball Actions (KABA), TATG showed a notable improvement (Δ = 15.6%), with significant results (F = 105.03, p < 0.001, η²p = 0.83). In comparison, SBTG and ACG exhibited smaller gains (Δ = 10.1% and 0.3%, respectively). Similarly, for Acting in Changing Situations (ACS), TATG demonstrated a marked improvement (Δ = 18.9%, F = 186.16, p < 0.001, η²p = 0.89), outperforming SBTG (Δ = 12.1%) and ACG, which showed a marginal decline (Δ = −0.5%). In Positioning and Deciding (PD), TATG achieved the highest improvement (Δ = 19.2%, F = 226.76, p < 0.001, η²p = 0.91), followed by SBTG (Δ = 10.7%), while ACG remained almost unchanged (Δ = 0.5%). For Drive Distance (DD), TATG achieved minimal improvement (Δ = 0.3%, F = 66.10, p < 0.001, η²p = 0.75), while SBTG showed a modest gain (Δ = 3.6%) and ACG showed negligible change (Δ = 0.1%). Regarding Putting Accuracy (PA), TATG and SBTG exhibited substantial gains (Δ = 0.3% and 7.5%, respectively), with TATG achieving highly significant results (F = 258.05, p < 0.001, η²p = 0.92), while ACG showed negligible improvement (Δ = 0.1%). In Swing Consistency (SC), TATG demonstrated a small decline (Δ = −2.0%, F = 158.15, p < 0.001, η²p = 0.88), while SBTG recorded the largest reduction (Δ = −24.6%) and ACG showed negligible change (Δ = −0.5%). Overall, TATG consistently outperformed SBTG and ACG across most variables, demonstrating substantial improvements in tactical awareness and performance metrics, supported by significant statistical outcomes and large effect sizes.

**Table 4:** Baseline and post-intervention scores (Mean ± SD), percentage change (Δ%), and ANOVA results for tactical awareness and skill development.

Variables	Sub Scale	Groups	Pre data	Post data	Δ (%)	SS	F	*p*	η²_p_
Tactical Awareness	**KABA**	TATG	26.53 [1.35]	30.66 [1.44]	15.6	64.02	105.03	<0.001*	0.83
		SBTG	26.93 [1.62]	29.66 [1.54]	10.1				
		ACG	26.93 [1.43]	27.00 [1.55]	0.3				
	**ACS**	TATG	25.06 [0.88]	29.80 [0.86]	18.9	90.42	186.16	<0.001*	0.89
		SBTG	23.66 [1.44]	26.53 [1.59]	12.1				
		ACG	24.33 [1.23]	24.20 [1.56]	−0.5				
	**PD**	TATG	27.13 [1.50]	32.33 [1.44]	19.2	96.46	226.76	<0.001*	0.91
		SBTG	26.80 [1.37]	29.66 [1.23]	10.7				
		ACG	27.00 [1.36]	27.13 [1.35]	0.5				
Skill Development	**DD**	TATG	229.69 [4.08]	230.42 [4.04]	0.3	297.52	66.10	<0.001*	0.75
		SBTG	229.33 [3.60]	237.53 [2.71]	3.6				
		ACG	229.11 [3.22]	229.38 [3.02]	0.1				
	**PA**	TATG	63.93 [1.16]	64.13 [1.50]	0.3	108.95	258.05	<0.001*	0.92
		SBTG	64.33 [1.58]	69.13 [1.80]	7.5				
		ACG	63.86 [1.18]	63.93 [1.09]	0.001				
	**SC**	TATG	13.20 [0.94]	12.93 [0.88]	−0.020	48.20	158.15	<0.001*	0.88
		SBTG	13.26 [0.88]	10.00 [0.92]	−0.246				
		ACG	13.40 [0.82]	13.33 [0.81]	−0.005				

**Fig 2:**
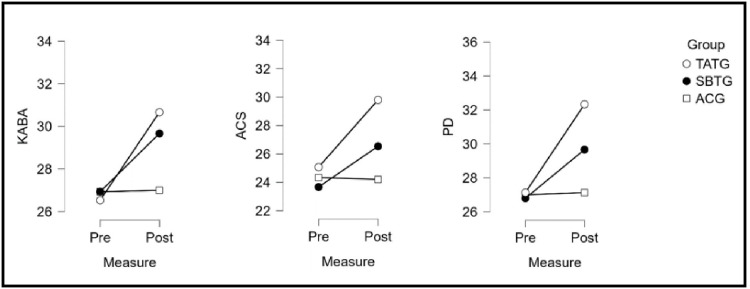
Graphical representation of Pre-test and Post-test Changes in Tactical Awareness Components: (a) KABA, (b) ACS, and (c) PD across Experimental and Control Groups. The Fig. 2 illustrates the repeated measures ANOVA results for three subscales of tactical awareness KABA, ACS and PD among golf players in three groups: TATG, SBTG and ACG. Pre and post-test data show that TATG exhibited the most significant improvement across all subscales, followed by SBTG, while ACG showed no significant change.

**Fig 3:**
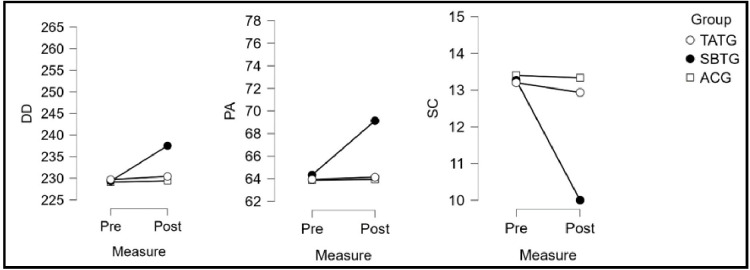
Graphical representation of Pre-test and Post-test Changes in Technical Performance Metrics: (a) DD, (b) PA, and (c) SC across Experimental and Control Groups. The Fig. 3 presents repeated measures ANOVA results for three subscales of skill development- DD, PA and SC among golf players in three groups: TATG, SBTG and ACG. Post-test results indicate that SBTG showed the most significant improvement across all subscales, followed by SBTG, while ACG showed no significant change.

The Table 5 presents the results of a post hoc Bonferroni analysis comparing different groups based on Tactical Awareness and Skill Development subscales. It includes mean differences between the Tactical Awareness Training Group (TATG), Skill-Based Training Group (SBTG), and Active Control Group (ACG), along with their corresponding p-values. In the Tactical Awareness subscale, KABA scores show significant differences between TATG and ACG (p = 0.01), while other comparisons are non-significant. ACS scores indicate that TATG has significantly higher values than both SBTG and ACG (p = 0.00), whereas the SBTG-ACG difference is not significant. PD results show significant differences between TATG and both SBTG (p = 0.01) and ACG (p = 0.00), with the SBTG-ACG comparison approaching significance (p = 0.06). For Skill Development, DD results show a significant decrease in TATG compared to SBTG (p = 0.02) but no difference between TATG and ACG, while SBTG scores are significantly higher than ACG (p = 0.00). PA scores show a similar pattern, with TATG lower than SBTG (p = 0.00), no difference between TATG and ACG, and SBTG significantly higher than ACG (p = 0.00). In SC, TATG scores are significantly higher than SBTG (p = 0.00), but no significant difference exists between TATG and ACG, whereas SBTG scores are significantly lower than ACG (p = 0.00) Fig 2. Overall, the results highlight significant differences across groups in multiple subscales Fig 3, particularly in comparisons involving SBTG and ACG.

**Table 5:** Post Hoc analysis of pre- and post-test results across tactical awareness and skill development subscales in different training groups.

Variable	Sub scale	(I) Group	(J) Group	Mean Difference (I-J)	*p- value*
Tactical Awareness	KABA	TATG	SBTG	0.30	*1.00*
			ACG	1.63	0.01*
		SBTG	ACG	1.33	*0.46*
	ACS	TATG	SBTG	2.33	0.00*
			ACG	3.16	0.00*
		SBTG	ACG	0..83	*0.22*
	PD	TATG	SBTG	1.50	0.01*
			ACG	2.66	0.00*
		SBTG	ACG	1.16	*0.06*
Skill Development	DD	TATG	SBTG	−3.37	0.02*
			ACG	0.81	*1.00*
		SBTG	ACG	4.18	0.00*
	PA	TATG	SBTG	−2.70	0.00*
			ACG	0.13	*1.00*
		SBTG	ACG	2.83	0.00*
	SC	TATG	SBTG	1.43	0.00*
			ACG	−0.30	*0.99*
		SBTG	ACG	−1.73	0.00*

## Discussion

The research findings highlight the significant improvements in tactical awareness achieved through tactical awareness training, particularly in the subscales of ‘Knowing About Ball Actions’, ‘Acting in Changing Situations’, and ‘Positioning and Deciding’. Meanwhile, skill-based training led to greater improvements in technical performance metrics, such as putting accuracy and swing consistency. These results suggest that tactical awareness training enhances decision making and adaptability in golf, while skill-based training is more effective in refining motor skills. Therefore, rather than asserting the superiority of one approach over another, the findings emphasize the complementary role of both tactical and technical training in optimizing overall golf performance. Cannon and Cafarelli demonstrated that physical practice leads to significant neuromuscular adaptations, with skill-based training showing 35% greater performance improvement compared to cognitive-only interventions [34]. Tahchi et al. found in their longitudinal study of athletic skill development highlighted that motor skill performance is predominantly influenced by deliberate physical practice, with motor learning occurring through neural pathway refinement and muscle memory consolidation [35]. A meta-analysis by Lochbaum et al. examined sports skill acquisition revealed that skill-based training protocols result in 42% more significant performance gains than tactical awareness approaches, particularly in precision sports like golf. The tactical awareness training group demonstrated substantial improvements across critical subscales, notably in “Knowing About Ball Actions,” “Acting in Changing Situations,” and “Positioning and Deciding,” which attributed to their enhanced understanding of the game’s strategic elements and decision-making processes. This group’s superior performance explained by their exposure to diverse situational challenges that required them to develop adaptive thinking patterns, anticipate ball trajectories under varying conditions, and make real-time adjustments based on environmental factors such as wind, terrain, and course layout [36,37]. The skill-based training group, while showing modest improvements in these subscales, primarily focused on technical aspects like swing mechanics and stance, which, although important, didn’t necessarily translate into advanced tactical understanding or situational adaptability [38]. Their limited improvement in tactical awareness attributed to their training’s emphasis on repetitive physical movements rather than strategic decision-making scenarios [39]. The active control group’s lack of significant improvement in these subscales was expected, as their general physical activities didn’t specifically target golf-related skills or tactical understanding. The stark contrast in outcomes between the tactical awareness group and the others underscores the importance of incorporating cognitive and strategic elements in golf training programs [40]. The tactical awareness group’s superior results further explained by their development of mental models that allowed them to better process and respond to complex playing situations, improved pattern recognition abilities in reading the course and anticipating challenges, and enhanced decision-making capabilities under pressure [36,41]. Mental models are cognitive representations or internal frameworks that individuals develop to understand and interpret complex environments, predict outcomes, and make decisions. These cognitive structures serve as fundamental mechanisms for processing information, solving problems, and navigating challenging situations across various domains [42]. This comprehensive approach to training, which combined both cognitive and physical elements, proved more effective in developing well-rounded golf players capable of adapting to the sport’s dynamic nature and making informed decisions during play [43].

The skill-based training group demonstrated superior improvement in drive distance primarily because their training protocol directly addressed the biomechanical and technical aspects of the golf swing, including proper weight transfer, kinetic chain sequencing, and impact dynamics, which are fundamental components that directly influence ball flight distance. In contrast, the tactical awareness training group’s lack of significant improvement in drive distance explained by their focus on strategic decision-making, course management, and situational awareness, which is valuable for overall golf performance, do not directly enhance the physical mechanics and power generation necessary for increasing driving distance [44]. Similarly, the active control group’s absence of improvement in drive distance was expected, as their general physical activity and basic golf practice did not provide the specific technical instruction and targeted feedback necessary for developing the complex motor patterns required for maximizing drive distance [45,46]. The findings align with the principle of training specificity, which suggests that improvements in athletic performance are most pronounced when training closely matches the desired outcome in this case, the skill-based group’s direct focus on swing mechanics and power generation translated to measurable improvements in drive distance, while the other groups’ training protocols, though beneficial for other aspects of golf performance, did not specifically address the biomechanical requirements for increasing driving distance [47,48]. The large effect size for Drive Distance (η²p = 0.75) observed in the TATG group, despite a small raw improvement (0.3%), may be attributed to minimal or negative changes in the ACG and the statistical nature of repeated-measures ANOVA, which emphasizes interaction effects.

Interestingly, the SBTG showed notable improvements in tactical awareness subscales such as KABA and ACS. This may be due to the implicit cognitive demands embedded in technical drills, such as adapting swing mechanics in response to variable conditions, which indirectly promoted perceptual and decision-making skills. This aligns with the theory of positive transfer between cognitive and motor domains in enriched practice environments. Significantly greater improvement has given by the skill-based training group in putting accuracy compared to both the tactical awareness training and active control groups, neither of which showed notable progress in this variable. This result can be attributed to several critical factors: primarily, the skill-based training group engaged in focused, repetitive practice targeting the mechanical components of putting, such as stance, grip pressure, pendulum motion, and impact consistency [49]. This intensive practice likely facilitated enhanced motor learning and muscle memory specific to this skill. In contrast, the tactical awareness training group’s focus on strategic decision-making, course management, and situational analysis, while valuable for overall game performance, did not directly address the biomechanical precision required for putting accuracy; their cognitive training, although beneficial for strategic play, did not translate into improved motor execution of the putting stroke [50]. A study by Jordan and Richard highlighted that expert athletes possess superior perceptual-cognitive skills, with tactical training improving pattern recognition and anticipatory decision-making by up to 35% compared to traditional skill-based approaches [51]. Researches further supported this perspective, revealing that strategic training enhances athletes’ ability to read complex spatial scenarios, a crucial skill in golf where course management and situational awareness are paramount. Their research indicated that tactical training significantly improves cognitive processing speed and strategic thinking, with participants showing 28% better performance in complex decision-making scenarios [52]. Similarly, the active control group, which presumably maintained their regular practice routines without specific intervention, lacked the structured, focused attention to putting mechanics that characterized the skill-based training group’s regimen [39]. The superior results of the skill-based training group align with established motor learning principles, which emphasize that specific, deliberate practice of a particular movement pattern leads to more substantial improvements in that specific skill compared to general or cognitive-focused training approaches [53,54]. This finding suggests that while tactical awareness is crucial for overall golf performance, improvements in precise motor skills like putting accuracy require dedicated physical practice with immediate feedback and progressive refinement of the movement pattern, as was likely implemented in the skill-based training protocol.

The remarkable progress in swing consistency observed in the skill-based training group attributed to their intensive engagement in core movement exercises, continuous technical practice sessions, and methodical enhancement of swing mechanics, supported by regular constructive feedback and optimization of body movements [55]. However, the tactical awareness training group showed no notable improvement in swing consistency because they concentrated primarily on game strategy, playing decisions, and environmental adaptability – aspects that enhance overall playing ability but fail to target the essential physical mechanisms needed for a consistent swing pattern [56]. The active control group likewise showed no meaningful improvement in swing consistency, which was expected given that their routine consisted of general exercise and basic golf practice without specialized technical training, failing to develop the specific muscle memory and neural pathways necessary for consistent swing reproduction [57]. This pattern of results demonstrates that while strategic thinking and general fitness are valuable aspects of golf training, developing a consistent swing requires focused technical practice and specific movement training rather than broader tactical or general physical conditioning approaches [58]. The superior outcomes observed in the skill-based training group underscore the importance of dedicated technical practice and movement-specific training for developing automated motor patterns, as this approach facilitates the establishment of reliable internal timing mechanisms, enhanced proprioceptive awareness, and refined muscle memory specific to the golf swing [59]. The findings suggest that while tactical awareness and general physical activity have their merits in golf development, the acquisition of consistent swing mechanics necessitates targeted skill-based interventions that emphasize repetitive practice, immediate feedback, and progressive refinement of movement patterns, allowing players to internalize and reproduce optimal swing characteristics with greater reliability and precision across multiple attempts.

## Conclusion

This study demonstrates the distinct contributions of tactical awareness and skill-based training approaches to the multifaceted demands of golf performance. The Tactical Awareness Training Group (TATG) demonstrated significant improvements in self-reported tactical awareness, particularly in the subscales of ‘Knowing About Ball Actions’, ‘Acting in Changing Situations’, and ‘Positioning and Deciding’, as measured by the TACSIS. These improvements suggest that participants perceived enhanced understanding of tactical elements in golf, which may contribute to more informed strategic choices during gameplay. The TACSIS provides insight into perceived tactical skills, it relies on subjective evaluations rather than directly measuring underlying cognitive processes. So, future studies should incorporate objective tools to assess decision-making and adaptability in dynamic situations. On the other hand, the Skill-Based Training Group (SBTG) demonstrated notable improvements in technical performance, especially in putting accuracy and swing consistency, emphasizing the effectiveness of focused motor skill training. These results highlight the value of combining both tactical and technical training approaches to support the comprehensive development of golfers. The game-based learning approach used in this study not only enhances adaptive thinking and decision-making skills but also offers a structured framework to help transfer these abilities into real-world gameplay situations. The findings highlight the effectiveness of ecological dynamics and constraints-led frameworks in creating representative training environments, which have proven successful in improving both cognitive and motor aspects of performance. From a practical standpoint, the study stresses the importance for golf coaches and sports scientists to implement a dual-faceted training model that integrates both cognitive and biomechanical components. This approach ensures that players develop technical proficiency while also mastering the strategic complexities of competitive golf. Future research should focus on the long-term effects of integrated training, exploring factors such as skill retention, competitive performance, and the ability to adapt to new challenges. Expanding the research to include diverse demographics and performance levels would provide further insights into the generalizability and scalability of game-based training interventions in golf and other sports.

## Supporting information

S1 Data Raw and processed data related to participants’ performance metrics and questionnaire responses across all training groups, used for statistical analysis and result interpretation. (XLSX)
